# Stability Indicating HPLC Method for the Determination of Fulvestrant in Pharmaceutical Formulation in Comparison with Linear Sweep Voltammetric Method

**Published:** 2016

**Authors:** Alptug Atila, Bilal Yilmaz, Yucel Kadioglu

**Affiliations:** *Department of Analytical Chemistry, Faculty of Pharmacy, Ataturk University, 25240, Erzurum, Turkey.*

**Keywords:** Fulvestrant, LSV, HPLC, Stability indicating, Validation

## Abstract

This paper describes two rapid, sensitive and specific methods for the determination of fulvestrant in pharmaceutical preparations by high performance liquid chromatography (HPLC) and linear sweep voltammetry (LSV). HPLC method was used to study the degradation behaviour. Fulvestrant was subjected to degradation under the conditions of hydrolysis (acid and alkali), oxidation (30% H_2_O_2_). The linearity was established over the concentration range of 5-50 m g mL^-1 ^for LSV and 0.5-20 m g mL^-1 ^for HPLC method. The intra- and inter-day relative standard deviation (RSD) was less than 3.96 and 3.07% for LSV and HPLC, respectively. Limits of quantification were determined as 5.0 and 0.50 m g mL^-1 ^for LSV and HPLC, respectively. No interference was found from tablet excipients at the selected assay conditions. The methods were applied for the quality control of commercial fulvestrant dosage form to quantify the drug and to check the formulation content uniformity.

## Introduction

**Figure 1:**
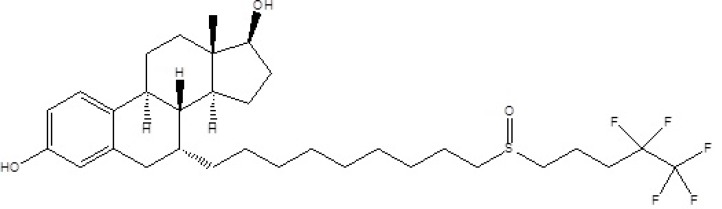
Chemical structure of the fulvestrant

Fulvestrant (Figure 1), 7-alpha-[9-(4,4,5,5,5-penta fluoropentylsulphinyl) nonyl]estra-1,3,5-(10)- triene-3,17-beta-diol, is a new estrogen receptor antagonist available for the treatment of hormone receptor-positive metastatic breast cancer in postmenopausal women (1). Although tamoxifen has been a great asset in the treatment of breast cancer, some of its features make it less than ideal (2, 3). Moreover, tamoxifen also increases the risk of endometrial cancer (4). For these reasons, there has been considerable interest in developing alternative hormonal treatments for breast cancer. 

Fulvestrant is an estrogen receptor antagonist with no known agonist effects; its mechanism of action works by down-regulating the estrogen receptor. It has a unique mode of action that offers the potential for continued hormonal treatment in patients and also offers potential therapeutic advantages over aromatase as it has been reported that it is similar to anastrozole in its primary efficacy. Fulvestrant has low aqueous solubility and has been developed as a long-acting, oil-based formulation for being used as a once-monthly intramuscular injection. This parenteral depot formulation provides adequate bioavailability and offers potential compliance advantages over existing breast cancer treatment. Intramuscular administration can offer sustained plasma drug concentration, and will also be less affected by vomiting and subsequent tablet loss than oral agents. 

Several research articles describing the pharmacology and pharmacokinetics of fulvestrant have been published, but very little information regarding its analytical methodology is available (5-8). There is a high-performance liquid chromatography and electrospray tandem mass spectrometry method for the determination of fulvestrant in rabbit plasma (9). 

The proposed method was influenced by the interference of endogenous substances and potential loss of drugs in the re-extraction procedure involving lengthy, tedious and time-consuming plasma sample preparation and extraction processes and requiring a sophisticated and expensive instrumentation. 

As a result of an extensive survey of literature, no LSV and HPLC methods are reported till date for determination of fulvestrant in pure and pharmaceutical dosage forms. The development of a new method capable of determining drug amount in pharmaceutical dosage forms is important. Electro-analytical techniques have been used for the determination of a wide range of drug compounds with the advantages that there are, in most instances, no need for derivatization and that these techniques are less sensitive to matrix effects than other analytical techniques. Additionally, application of electrochemistry includes the determination of electrode mechanism. Redox properties of drugs can give insights into their metabolic fate or their in vivo redox processes or pharmacological activity (10-13). Despite the analytical importance of the electrochemical behaviour and oxidation mechanism of fulvestrant, no report has been published on the voltammetric study of the electrochemical oxidation of fulvestrant in non-aqueous media. It is well known that the experimental and instrumental parameters directly affect the electrochemical process and the voltammetric response of drugs. Consequently, it would be interesting to investigate the oxidation process of fulvestrant in aprotic media. 

Therefore, this paper describes a new LSV and HPLC methods for the determination of fulvestrant. The LSV method was aimed at developing an easy and rapid assay method for fulvestrant without any time consuming sample preparation steps for routine analysis. HPLC method was attempted to demonstrate the utility of UV detection for the determination of fulvestrant with simple sample preparation and reasonable analysis time with high precision. 

In both proposed methods, there is no need to extract the drug from the formulation excipient matrix thereby decreasing the error in quantization. Formulation samples can be directly used after dissolution and filtration. The developed methods were used to determine the total drug content in commercially available injectable solution of fulvestrant. 

Also, the present study describes, for the first time, the development and validation of a stability-indicating HPLC method for stability evaluation and quantitative determination of fulvestrant in the presence of its degradation products.

## Experimental

*Chemicals and Reagents*

Fulvestrant was obtained from Astra Zeneca (Istanbul, Turkey). Acetonitrile (Fluka for HPLC analysis) was purified by drying with calcium hydride, followed by distillation from phosphorus pentoxide. After the purification in order to eliminate its water content as much as possible, it was kept over molecular sieves (3Å, Merck). Lithium perchlorate (LiClO_4_), methanol and orthophosphoric acid were purchased from Fluka (Buchs, Switzerland). Faslodex injectable solution was obtained from pharmacy (Erzurum, Turkey). 

*Voltammetric and chromatographic system*

Electrochemical experiments were performed on a Gamry Potentiostat Interface 1000 controlled with software PHE 200 and PV 220. All measurements were carried out in a single-compartment electrochemical cell with a standard three-electrode arrangement. A platinum disk with an area of 0.72 cm^2^ and a platinum wire were used as the working and the counter electrodes, respectively. The working electrode was successively polished with 1.0, 0.3 and 0.05 µM alumina slurries (Buehler) on microcloth pads (Buehler). After each polishing, the electrode was washed with water and sonicated for 10 min in acetonitrile. Then, it was immersed into a hot piranha solution (3:1, H_2_SO_4_, 30% H_2_O_2_) for 10 min, and rinsed copiously with water. *Caution: Piranha is a vigorous oxidant and should be used with extreme caution! *All potentials were reported versus Ag/AgCl/KCl (3.0 M) reference electrode (BAS Model MF-2078) at room temperature. The electrolyte solutions were degassed with purified nitrogen for 10 min before each experiment and bubbled with nitrogen during the experiment. 

Chromatographic analysis was carried out on an Agilent 1200 series HPLC system, consisting of a degasser, quaternary pump, autosampler, and variable wavelength UV detector units. The reversed-phase ACE C_18_ analytical column (250 mm × 4.6 mm I.D., 5 μM) was used in chromatographic separation. The column and the HPLC system were kept in ambient conditions. The mobile phase was a mixture of 1% orthophosphoric acid -methanol (80:20, v/v) prepared at a flow rate of 1.0 mL min^-1^ and the injection volume was 10 μL. 

*Preparation of the standard and quality control solutions*

For the LSV method, the stock standard solution of fulvestrant was prepared in 0.1 M LiClO_4_/acetonitrile to a concentration of 100 m g mL^-1^. For the HPLC method, the stock solution of fulvestrant was prepared in methanol solution to a concentration of m g mL^-1^. Standard solutions were prepared as 5-50 µg mL^-1 ^(5, 10, 15, 20, 30, 40 and 50 m g mL^-1^) for LSV and 0.5-20 m g mL^-1^ (0.5, 1, 2, 5, 10, 15 and 20 m g mL^-1^) for the HPLC method. The quality control (QC) samples were prepared by adding aliquots of standard working solution of fulvestrant to final concentrations of 7.5, 25 and 45 m g mL^-1^ for the LSV and 0.75, 8 and 18 m g mL^-1 ^for the HPLC method.

*Procedure for pharmaceutical preparations*

For the LSV method, an adequate amount of Faslodex injectable solution, claimed to contain 250 mg fulvestrant per 5mL of the solution, was dissolved in 50 mL of 0.1 M LiClO_4_/acetonitrile then the flask was sonicated for 10 min at room temperature. The flask was filled to volume with 0.1 M LiClO_4_/acetonitrile. The resulting solutions in both the cases were filtered through Whatman filter paper no 42 and suitably diluted to get final concentration within the limits of linearity for the respective proposed method. For the HPLC method, an appropriate volume of filtrate was diluted further with methanol so that the concentration of fulvestrant in the final solution was within the working range, and then analysed by HPLC.

*Data analysis*

All statistical calculations were performed with the Statistical Product and Service Solutions (SPSS) for Windows, version 10.0. Correlations were considered statistically significant if calculated P values were 0.05 or less.

**Figure 2:**
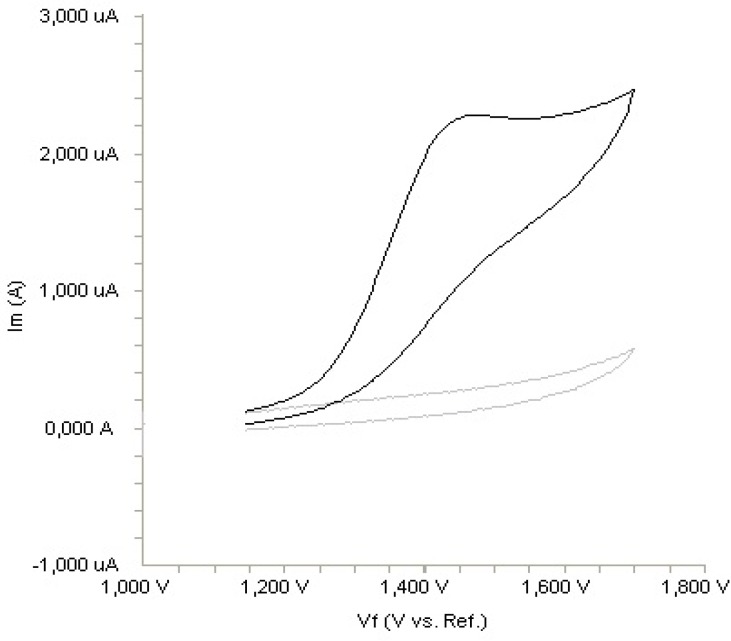
Cyclic voltammogram for the oxidation of 20 µg mL^-1^ fulvestrant in acetonitrile containing 0.1 M LiClO_4_ at Pt disk electrode, scan rate: 0.1 V s^-^

**Figure 3.**
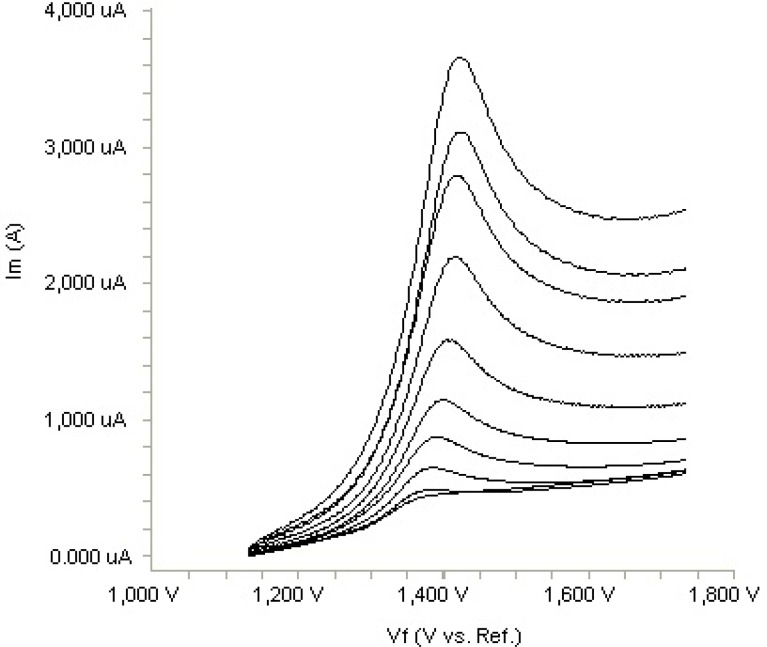
Linear sweep voltammograms for the oxidation of 20 µ

**Figure 4 (a-c):**
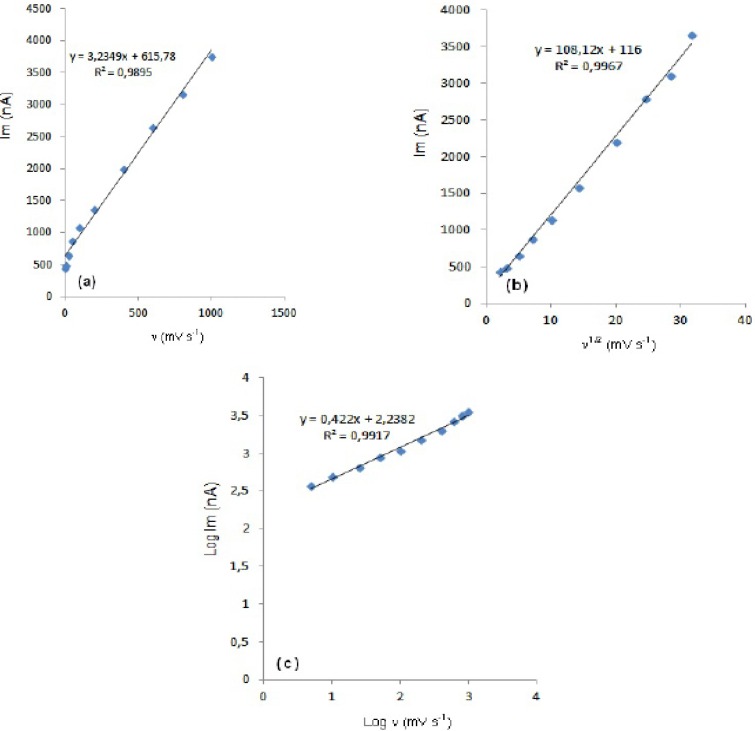
Dependence of peak current on the scan rate (20 µg mL^-1^).

**Figure 5:**
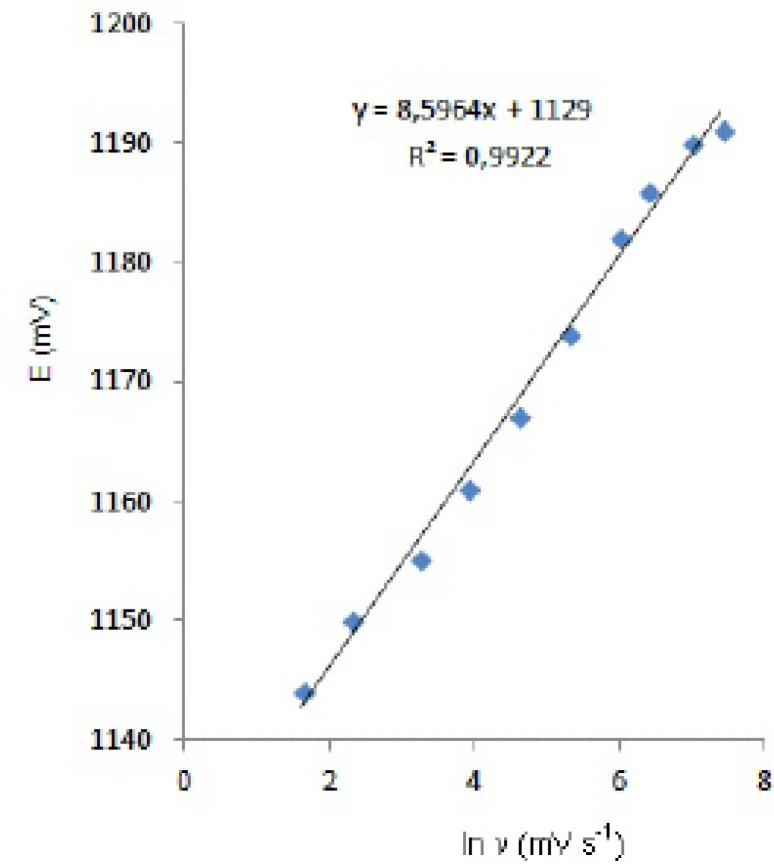
Dependence of anodic peak potentials of voltammetric peak for the oxidation of 20 µg mL^-1 ^fulvestrant on the scan rate.

**Figure 6:**
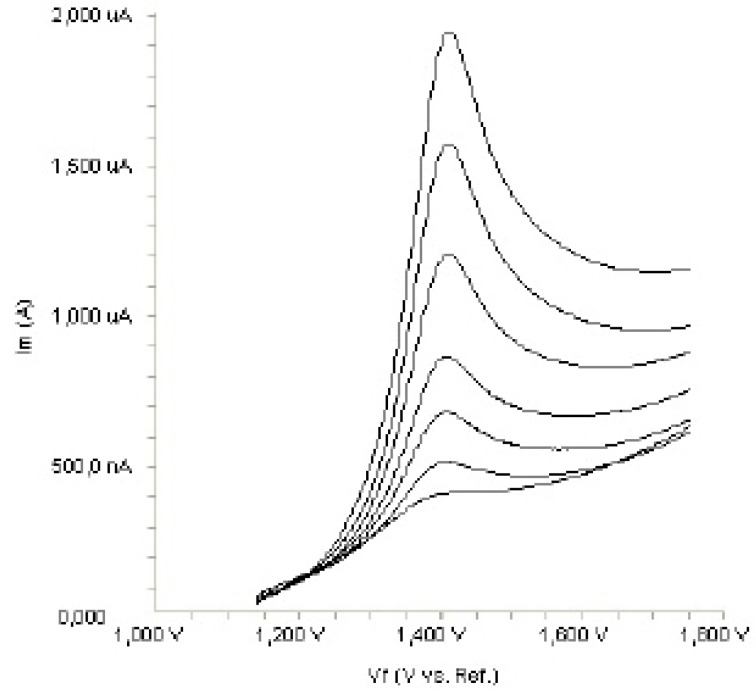
Linear sweep voltammograms for different concentrations of fulvestrant in acetonitrile solution containing 0.1 M LiCIO_4_ (5, 10, 15, 20, 30, 40 and 50 µg mL^-^

**Figure 7:**
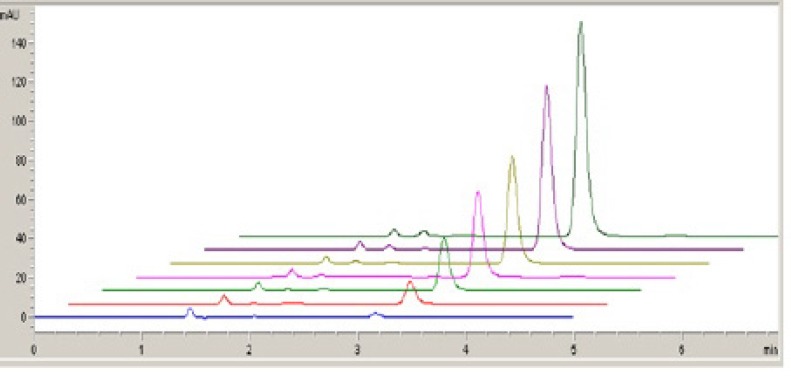
HPLC chromatograms of fulvestrant (0.5, 1, 2, 5, 10, 15 and 20 m g mL^-1^).

**Figure 8:**
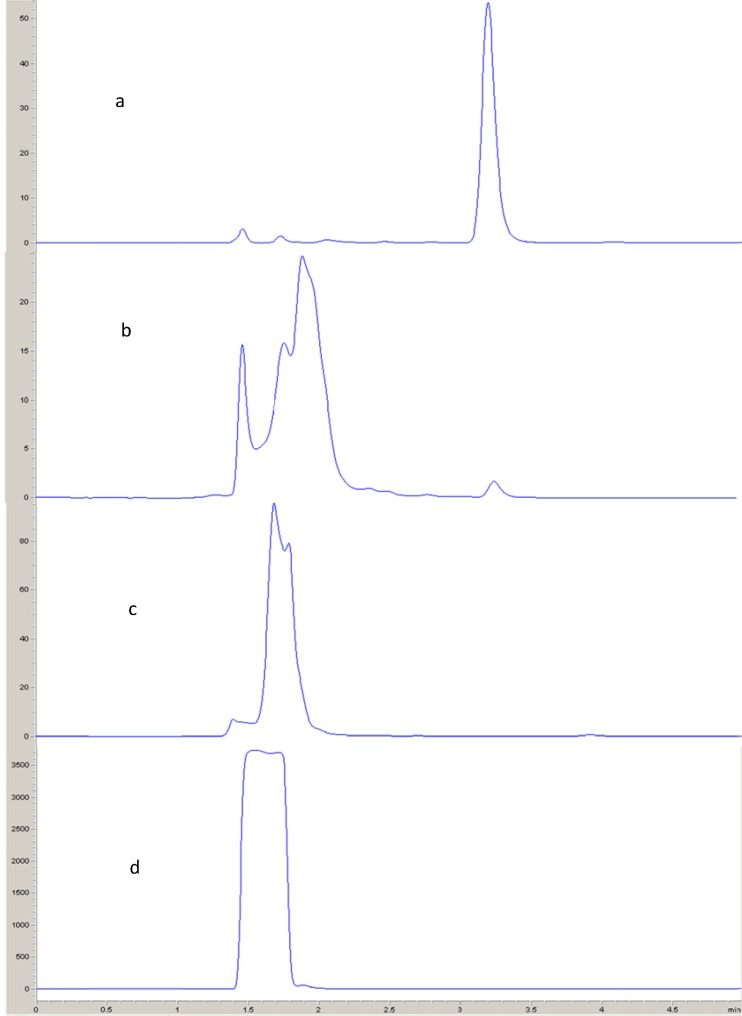
HPLC chromatograms of fulvestrant (a) 0.1 M NaOH, (b) 0.1 M HCl and (c) 0.1 M H_2_O_2_.

**Table 1:** Linearity of fulvestrant

**Method**	**Range (µg mL** ^-1^ **)**	**LR**	**R**	** LOD (µg mL** ^-1^ **)**	** LOQ (µg mL** ^-1^ **)**
LSV	5 - 50	y = 23.424x + 139.74	0.9992	1.52	5.00
HPLC	0.5 - 20	y = 36.903x – 3.0597	0.9999	0.152	0.50

^a^Based on three calibration curves, LR: linear regression, R: Coefficient of correlation, x: fulvestrant concentration (µg mL^-1^),

LOD: Limit of detection, LOQ: limit of quantification.

**Table 2:** Precision and accuracy of fulvestrant

**Method**		**Added** ** (µg mL** ^-1^ **)**		**Intra-day**						**Inter-day**					
				**Found±SD** ^a^		**Precision** **% RSD** b		**Accuracy** c		**Found±SDa**		**Precision** **% RSD** b		**Accuracy** c	
LSV		7.5		7.38 ± 0.12		1.58		- 1.55		7.47 ± 0.10		1.38		- 0.44	
		25		25.00 ± 0.89		3.58		0.04		24.83 ± 0.98		3.96		- 0.67	
		45		45.33 ± 1.03		2.28		0.74		45.67 ± 1.03		2.26		1.48	
HPLC		0.75		0.73 ± 0.01		1.37		- 2.66		0.76 ± 0.02		2.63		1.33	
		8		8.13 ± 0.25		3.07		1.63		7.91 ± 0.22		2.78		- 1.13	
		18		17.71 ± 0.44		2.48		- 1.61		17.84 ± 0.39		2.19		- 0.89	

SD: Standard deviation of six replicate determinations,

b RSD: Relative standard deviation, Average of six replicatedeterminations,

c Accuracy: (%relative error) (found-added)/addedx100

**Table 3:** Recovery of fulvestrant in pharmaceutical preparation

**Commercial Preparation**		**Method**		**n**		**Found(mg) Mean±SD**		**Recovery**		**% RSD** a		**Confidence Interval**	
Faslodex injection (250 mg/5 mL)		LSV		6		254.5 ± 2.30		101.8		0.90		247.9 - 252.6	
		HPLC		6		252.5 ± 2.05		102.0		0.81		248.2 - 252.7

SD: Standard deviation of six replicate determinations, RSD: Relative standard deviation

a , Average of six replicate determinations

**Table 4:** The results of analyses of fulvestrant by a different analyst^a^

**Method**		**Added (µg mL** ^-1^ **)**		**Found (µg mL** ^-1^ **)** **Mean±SD**		**% Recovery**		**% RSD** a	
LSV		5		5.1 ± 0.18		102.0		3.53	
		15		14.8 ± 0.25		98.7		1.69
		35		35.2 ± 1.67		100.6		4.74
HPLC		3		3.1 ± 0.16		103.3		5.16	
		9		8.8 ± 0.28		97.8		3.18
		15		15.6 ± 0.71		104.0		4.55

a Mean measurements of six replicate determinations

**Table 5:** Comparison of the proposed and reported methods for determination of fulvestrant

**Parameters**	**LSV**	**HPLC**	**Reported** **Method ** ^21^
Mean (recovery %)	101.8	102.0	100.9
SD	1.79	2.48	4.31
%RSD	1.76	2.43	4.27
Variance	3.20	6.16	
F-test	3.18	3.78	

SD: Standard deviation of six replicate determinations, RSD: relative standard deviation,

Theoretical values at P=0.05, Ho hypothesis: no statistically significant difference exists between four methods,

F_t_ >F_c_: Ho hypothesis is accepted (P > 0.05)

## Results and discussion

*Method development and optimization*

The electrochemical behaviour of fulvestrant was investigated at the Pt disc electrode in acetonitrile solution containing 0.1 M LiClO_4 _as the supporting electrolyte by using cyclic voltammetry (CV). Figure 2. shows a typical cyclic voltammogram of 20 m g mL^-1 ^fulvestrant recorded under these conditions for the scan rate of 0.1 V s^-1^. In the anodic sweep, an oxidation peak is seen at about potential of 1.4 V. Upon reversing the potential scan, no reduction peak corresponding to this oxidation wave is observed, indicating the irreversible nature of the electrode reactions. 

In order to gain a deeper insight into the voltammetric waves, the effect of scan rate on the anodic peak currents (I_m_) and peak potentials (E_p_) was studied in the range of 0.01-1 V s^-1^ of the potential scan rates in acetonitrile solution containing 20 m g mL^-1 ^concentration of fulvestrant ( 3.). The representative linear sweep voltammograms obtained at Pt electrode for 20 m g mL^-1 ^concentration of fulvestrant display straight lines with 0.42 slope (Figure 4c), which are close to theoretical value of 0.5 expected for an ideal diffusion-controlled electrode process.^14^ log I_m_-log ν curve is more eligible for this aim, therefore, a diffusional process for peak should be considered. These results suggest that the redox species are diffusing freely from the solution and not precipitating onto the electrode surface. The reason for this behaviour may be due to the solubility of the intermediate species in acetonitrile or poor adherence of products on the electrode surface.As shown in Figure 3. the oxidation peak potential (E_pa_) for peaks shift toward more positive values with increasing scan rate. The relationship between the peak potential and scan rate is described by the following equation,

$E_{\mathrm{pa}}=E^{0'}+\frac{\mathrm{RT}}{\left[\left(1-\alpha \right)n_{\alpha }F\right]0.78}+\ln+\left(D^{\frac{1}{2}}k_{s}^{-1}\right)-\frac{0.5\mathrm{lnRT}}{\left[\left(1-\alpha \right)n_{\alpha }F\right]}+\frac{\mathrm{RT}}{\left[\left(1-\alpha \right)n_{\alpha }F\right]}/2\mathrm{lnv}$

and from the variation of peak potential with scan rate αn_a_ can be determined, where α is the transfer coefficient and n_a _is the number of electrons transferred in the rate determining step. According to this equation, the plots of the peak potentials versus ln ν for oxidation peak show linear relationship (Figure 5). The slope indicates the value of αn_a_ is 0.75 for peak. Also, this value obtained indicates the total irreversibility of the electron transfer processes. This result shows that the chemical step is a fast following reaction coupled to a charge transfer

During HPLC method development, different organic solvents were tested as mobile phase. The best peak was achieved with the mixture of 1% orthophosphoric acid-methanol (80:20, v/v). Chromatographic separation was achieved with reverse phase C_18_ analytical column. When the different wavelength was investigated with UV detection, the best chromatogram for fulvestrant was obtained at 243 nm wavelength. 

*Method validation*

To ensure the optimization of the methods in light of the standardization rules, we developed these methods along with the process of validation. The assay methods were evaluated through the determination of specificity, linearity, precision, ac­curacy, limit of detection, limit of quantification, recovery and the stability effect was investigated by analysing the pure fulvestrant solution and drug sample (15).

*Specificity*

All the solutions were scanned from 1.0 to 1.7 V and checked for change in the peaks at respective potentials (Figure 6). 

In a separate study, the specificity of the method was investigated by observing interferences between the fulvestrant and excipients. The retention time of fulvestrant in HPLC method was approximately 3.1 min with good peak shape (Figure 7).

*Linearity*

For LSV and HPLC measurements, the solutions were prepared by dilution of the stock solution of fulvestrant to reach a concentration range of 5-50 m g mL^-1^ (5, 10, 15, 20, 30, 40 and 50 m g mL^-1^) and 0.5-20 m g mL^-1^ (0.5, 1, 2, 5, 10, 15 and 20 m g mL^-1^), respectively. Calibration curves were constructed for fulvestrant standard by plotting the concentration of fulvestrant versus voltammogram and peak area response. The calibration curve constructed was evaluated by its correlation coefficient. The correlation coefficient (*r*) of all the calibration curves were consistently greater than 0.99. The regression equations were calculated from the calibration graphs, along with the standard deviations of the slope and intercept on the ordinate. The results are shown in Table 1.

*Precision and accuracy*

The precision of the LSV and HPLC methods was determined by repeatability (intra-day) and intermediate precision (inter-day). Repeatability was evaluated by analysing QC samples six times per day, at three different concentrations which were QC samples. The intermediate precision was evaluated by analysing the same samples once daily for two days. The RSD of the predicted concentrations from the regression equation was taken as precision (16-19). The accuracy of this analytic method was assessed as the percentage relative error. For all the concentrations studied, intra- and inter-day relative standard deviation values were £ 2.66%. These results were given in Table 2.

*Limits of detection (LOD) and quantification (LOQ)*

For LSV measurements, LOD and LOQ of the fulvestrant were determined using calibration standards. The LOD and LOQ values were calculated as 3.3 *σ*/*S *and 10 *σ*/*S*, respectively, where *S *is the slope of the calibration curve and *σ *is the standard deviation of *y*-intercept of regression equation (*n = *6) (20).

For HPLC measurements, the LOD and LOQ of the fulvestrant were determined by injecting progressively low concentration of the standard solution under the chromatographic conditions. The lowest concentrations assayed where the signal/noise ratio was at least 10:1, this concentration was regarded as LOQ. The LOD was defined as a signal/noise ratio of 3:1. The LOD and LOQ for LSV were 1.52 and 5.0 m g mL^-1^, for HPLC 0.152 and 0.50 m g mL^-1^, respectively. Among the two methods, HPLC is more sensitive than LSV.

*Recovery*

To determine the accuracy of the LSV and HPLC methods and to study the interference of formulation additives, the recovery was checked as three different concentration levels. Analytical recovery experiments were performed by adding the known amount of pure drugs to pre-analyzed samples of commercial dosage form. The recovery values were calculated by comparing the concentration obtained from the spiked samples with actual added concentrations. These values are also listed in Table 3.

*Ruggedness*

In this study, the LSV and HPLC determination of fulvestrant were carried out by a different analyst in the same instrument with the same standard (Table 4). The results showed no statistical differences between different operators suggesting that the developed method was rugged.

*Stability*

Stability studies indicated that the samples were stable when kept at room temperature, +4 ^0 ^C and -20 ^0 ^C refrigeration temperature for 24 h (short-term) and refrigerated at +4 and -20 ^0 ^C for 72 h (long-term). There was no significant change in the analysis over a period of 72 h. The mean RSD between peak areas for the samples stored under refrigeration (4 ± 1 ° C), at room temperature (25 ± 1 ° C) and refrigeration (-20 ± 1 ° C) were found to be 1.47%, 1.78% and 1.92%, respectively, suggesting that the drug solution can be stored without any degradation over the studied time interval.

Also, The ICH guideline entitled stability testing of drug substances and products requires the stress testing to be carried out to elucidate the inherent stability characteristics of the active substance, and provide a rapid identification of differences that might result from changes in the manufacturing processes or source sample (19). Susceptibilities to acid, alkali and oxidation hydrolysis stability are the required tests. 

*Acid and alkali hydrolysis*

Aliquot of 0.2 mL of fulvestrant solution (50 m g mL^-1^) was transferred to a small rounded flask. The solution was mixed with 0.8 mL of 0.1 N hydrochloric acid, or 0.1 N sodium hydroxide. The prepared solutions were subjected to reflux for 2 h in a boiling water bath. The samples were cooled to room temperature (25 ± 5 ° C), neutralized with an amount of acid or base equivalent to that of the previously added. From the resulting neutral solution, 10 μL was injected into the HPLC system (Figure 8). 

*Oxidation*

0.2 mL of fulvestrant solution (50 m g mL^-1^) was transferred to rounded flask. The contents were then mixed with 0.8 mL of 30% hydrogen peroxide solution, and the reaction mixture was allowed to proceed at room temperature (25 ± 5 ° C) for 2 h with intermittent shaking. A volume of 10 μL was injected into the HPLC system (Figure 8).

*Comparison of the methods*

The first paper related to electrochemical investigation of the fulvestrant has been reported by Dogan Topal and Ozkan (21). In this paper, the electrochemical oxidation of the fulvestrant has been studied by means of cyclic voltammetry and differential pulse voltammetry in pH 4.80 acetate buffer (30% ethanol) at a modified pencil graphite electrode, and it was reported that the drug exhibited an irreversible and diffusion-controlled oxidation peak. According to the molecular structure, literature knowledge and the obtained results, the oxidation mechanism of the fulvestrant may be postulated by an initial oxidation with two electrons and the conversion of hydroxyl group to quinone, which was electro-active in both acidic and basic media (22, 23). Also, LSV and HPLC methods were applied for the determination of fulvestrant in Faslodex injectable solution. The results show the high reliability and reproducibility of two methods. The results were statistically compared using the F-test. At 95% confidence level, the calculated *F*-values do not exceed the theoretical values (Table 5) Therefore, there is no significant difference between LSV and HPLC method. 

Also, the suggested LSV and HPLC methods were compared with the reported differential pulse voltammetry (21). There was no significant difference between the three methods with respect to mean values and standard deviations at the 95% confidence level (Table 5). Therefore, it is suggested that the two methods are equally applicable*.*

## Conclusion

In the present work, two new methods have been developed and validated for routine determination of fulvestrant in pharmaceutical preparations. Linearity range, precision, accuracy, LOD and LOQ are suitable for the quantification of fulvestrant in pharmaceutical preparations. The sample recoveries in three formulations were in good agreement with their respective label claims. No extraction procedure is involved. According to the statistical comparison of the results there is no significant difference between LSV and HPLC methods (Table 5). 

The proposed methods can be used for the routine quality control analysis of fulvestrant in pharmaceutical preparations in a total time of 5 min.
